# Effect of Purified Resveratrol Butyrate Ester Monomers against Hypertension after Maternal High-Fructose Intake in Adult Offspring

**DOI:** 10.3390/nu16183132

**Published:** 2024-09-17

**Authors:** You-Lin Tain, Chih-Yao Hou, Hong-Tai Tzeng, Shu-Fen Lin, Guo-Ping Chang-Chien, Wei-Chia Lee, Kay L. H. Wu, Hong-Ren Yu, Julie Y. H. Chan, Chien-Ning Hsu

**Affiliations:** 1 Department of Pediatrics, Kaohsiung Chang Gung Memorial Hospital, Kaohsiung City 833, Taiwan; tainyl@cgmh.org.tw (Y.-L.T.); yuu2004taiwan@gmail.com (H.-R.Y.); 2 Institute for Translational Research in Biomedicine, Kaohsiung Chang Gung Memorial Hospital, Kaohsiung City 833, Taiwan; htay11@cgmh.org.tw (H.-T.T.); klhwu@cgmh.org.tw (K.L.H.W.); jchan@cgmh.org.tw (J.Y.H.C.); 3 College of Medicine, Chang Gung University, Taoyuan 330, Taiwan; 4 Department of Seafood Science, National Kaohsiung University of Science and Technology, Kaohsiung City 811, Taiwan; chihyaohou@webmail.nkmu.edu.tw; 5 Super Micro Mass Research and Technology Center, Cheng Shiu University, Kaohsiung City 833, Taiwan; linsufan2003@csu.edu.tw (S.-F.L.); guoping@csu.edu.tw (G.-P.C.-C.); 6 Institute of Environmental Toxin and Emerging-Contaminant, Cheng Shiu University, Kaohsiung City 833, Taiwan; 7 Center for Environmental Toxin and Emerging-Contaminant Research, Cheng Shiu University, Kaohsiung City 833, Taiwan; 8 Department of Urology, Kaohsiung Chang Gung Memorial Hospital and Chang Gung University College of Medicine, Kaohsiung City 833, Taiwan; dinor666@ms32.hinet.net; 9 Department of Pharmacy, Kaohsiung Chang Gung Memorial Hospital, Kaohsiung City 833, Taiwan; 10 School of Pharmacy, Kaohsiung Medical University, Kaohsiung City 807, Taiwan

**Keywords:** nitric oxide, trimethylamine-*N*-oxide, developmental origins of health and disease (DOHaD), hypertension, gut microbiota, resveratrol, short-chain fatty acid, fructose

## Abstract

Background: Offspring hypertension arising from adverse maternal conditions can be mitigated through dietary nutritional supplementation, including resveratrol. Previously, we identified derivatives of resveratrol butyrate ester (RBE), specifically 3,4′-di-O-butanoylresveratrol (ED2) and 3-O-butanoylresveratrol (ED4), demonstrating their superior antioxidant capabilities compared to RBE itself. This study sought to assess the protective impact of maternal supplementation with ED2 or ED4 on offspring hypertension in a rat model subjected to a high-fructose (HF) diet during pregnancy and lactation. Methods: Female Sprague–Dawley rats were distributed into distinct dietary groups throughout pregnancy and lactation: (1) standard chow; (2) HF diet (60%); (3) HF diet supplemented with ED2 (25 mg/L); and (4) HF diet supplemented with ED4 (25 mg/L). Male offspring were euthanized at the age of 12 weeks. Results: The maternal HF diet induced hypertension in the offspring, which was mitigated by perinatal supplementation with either ED2 or ED4. These protective effects were attributed to the antioxidant properties of ED2 and ED4, resulting in an increased availability of nitric oxide (NO). Additionally, supplementation with ED2 was connected to an increased abundance of *Bifidobacterium* and *Clostridium* genera, which was accompanied by a decrease in *Angelakisella* and *Christensenella*. On the other hand, ED4 supplementation shielded rat offspring from hypertension by elevating concentrations of short-chain fatty acids (SCFAs) and their receptors while reducing trimethylamine-*N*-oxide (TMAO) levels. Conclusions: These findings highlight the potential of purified RBE monomers, ED2 and ED4, as preventive measures against hypertension resulting from a maternal high-fructose diet. Further research is warranted to explore their clinical applications based on these promising results.

## 1. Introduction

In recent decades, the replacement of sucrose with high-fructose corn syrup (HFCS) has significantly increased fructose consumption, now averaging about 7.5% of total energy intake [1]. This rise in HFCS consumption in the US has coincided with a surge in obesity rates [2]. Dietary fructose and beverages sweetened with fructose have been linked to heightened risk factors for cardiovascular disease (CVD), including obesity and metabolic syndrome [3,4]. Despite these associations, fructose intake among women, regardless of pregnancy status, remains above recommended levels [5]. Although high dietary fructose intake during pregnancy is associated with adverse pregnancy outcomes, its long-term effects on offspring remain poorly understood [6]. Considering the significant number of infants born to mothers with excessive fructose consumption, it is crucial to investigate and clarify its potential impact on the long-term health of these children.

Hypertension, a prevalent cardiovascular condition, is a significant contributor to global mortality [7]. Maternal malnutrition in humans can adversely affect kidney development, resulting in a reduced number of nephrons and an increased risk of hypertension [8]. Animal studies have demonstrated that high-fructose diets consumed by mothers during pregnancy and lactation can program kidney development, leading to hypertension in their adult offspring across various animal models [9,10,11]. Feeding mother mice a diet containing 20% fructose during pregnancy and lactation led to the development of hypertension in their offspring across multiple generations by 8 months of age [9]. Additionally, another study found that a maternal diet comprising 60% fructose induced kidney programming, resulting in hypertension in the adult offspring by 12 weeks [10].

The underlying mechanisms include heightened deficient nitric oxide (NO), gut microbiota imbalances, aberrant renin–angiotensin system activity, inflammation, and disruptions in nutrient sensing [9,10,11]. However, growing evidence suggests that early-life interventions targeting these mechanisms may offer protection against hypertension in adult offspring. For example, maternal melatonin treatment at a concentration of 0.01% in drinking water during gestation and lactation has been shown to counteract the effects of a high-fructose diet with one study demonstrating that it prevented hypertension in offspring by increasing NO levels in their kidneys [12].

Resveratrol, a phenolic phytochemical belonging to stilbene family, is renowned for its myriad of health benefits [13]. Its positive effects are primarily attributed to its antioxidant, anti-inflammatory, anti-hyperlipidemia, immune-modulating, augmentation of NO, and prebiotic properties [14,15]. Prior work suggests that resveratrol could offer significant benefits and protection in addressing hypertension with developmental origins [13,16]. However, a major challenge to its clinical application is its limited availability [17].

Recent studies highlight the health benefits of short-chain fatty acids (SCFAs), which are produced by gut microbial fermentation of dietary fiber [18]. We previously esterified resveratrol with SCFAs to enhance its bioactivity and antioxidant capacity [19,20]. We introduced novel resveratrol butyrate esters (RBEs) with the composition containing RBE monoester, RBE diester, and RBE triester [19,20]. Within the obtained ester derivatives, the primary compounds in the RBE complex were 3,4′-di-O-butanoylresveratrol (ED2) and 3-O-butanoylresveratrol (ED4). Notably, both ED2 and ED4 demonstrate superior antioxidant activity compared to other components in the RBEs [19,20].

In recent studies, we illustrated that RBEs exhibit prebiotic properties by promoting the growth of probiotics and influencing the production of microbial metabolites, including SCFAs and trimethylamine-*N*-oxide (TMAO) [21,22]. Although RBEs have shown a BP-lowering effect in a juvenile CKD rat model [22], their potential in improving maternal high-fructose diet-induced offspring hypertension has not been investigated and remains incompletely understood. Given that ED2 and ED4 possess higher antioxidant capacities than resveratrol, we aim to evaluate the efficacy of these two RBE components in mitigating offspring hypertension and to elucidate their protective mechanisms in a rat model with a maternal diet high in fructose.

## 2. Materials and Methods

### 2.1. Preparation of Resveratrol Butyrate Esters

Prepared using our previously described protocol [19,20], the RBE mixture was synthesized by combining resveratrol (TCI-SCT, Shanghai, China) with butyrate (ACROS, Morris Plains, NJ, USA) in tetrahydrofuran. Subsequently, *N*-ethyl-N^0^-(3-dimethylaminopropyl) carbodiimide (Sigma-Aldrich, Saint Louis, MO, USA) and 4-dimethylaminopyridine (Sigma-Aldrich) were introduced. The esterification reaction was performed in darkness for 48 h. After completion, distilled water was added to the reaction mixture, which was then filtered to isolate the precipitated RBE mixture and stored at −20 °C.

Following our established protocol [19], the RBE mixture underwent purification. Except for resveratrol, five distinct ester derivatives were isolated from silica gel: ED2 (3,4′-di-O-butanoylresveratrol, 18.8%), ED4 (3-O-butanoylresveratrol, 35.7%), ED5 (4′-O-butanoylresveratrol, 4.4%), ED6 (3,5,4′-tri-O-butanoylresveratrol, 1.5%), and ED7 (3,5-di-O-butanoylresveratrol, 0.7%) [19]. Subsequently, ED2 and ED4 were purified using acetone crystallization and employed in the subsequent experiments.

### 2.2. Animal Model

This study was granted an animal license 2022011001 by the Institutional Animal Ethics Committee and conformed to the ARRIVE guidelines. In the case of the experimental groups, mother rats were fed either a standard laboratory rat chow or a 60% HF diet from the time of mating until postnatal day 21, at which point the offspring were segregated into groups based on their respective litters. ED2 or ED4 was administered to dams at the dose of 25 mg/L in drinking water throughout pregnancy and lactation periods. A total of 32 male offspring were divided into four groups: group ND (normal diet), group HF (high-fructose diet), group HFED2 (high-fructose diet plus ED2 supplementation), and group HFED4 (high-fructose diet plus ED4 supplementation), n = 8 for each group. The dosage and route of ED2 and ED4 were selected based on prior rodent studies [22]. The research took place in an AAALAC-accredited animal facility, focusing exclusively on male offspring due to their greater susceptibility to hypertension at an earlier age [23].

BP was measured using the Kent Scientific CODA system (Torrington, CT, USA) in offspring from week 3 to 12 [22]. Fecal samples were collected and frozen at −20 °C. At 12 weeks of age, the rats were euthanized, and heparinized blood samples were collected and stored in a freezer. The kidneys were excised, and the medulla and cortex were dissected, immediately snap-frozen, and subsequently stored at −80 °C.

### 2.3. NO Pathway

Nitric oxide synthases (NOS) use arginine as a substrate to produce nitric oxide but can be inhibited by asymmetric and symmetric dimethylarginine (ADMA and SDMA). We measured these plasma NO parameters using HPLC (HP series 1100, Agilent Technologies Inc., Santa Clara, CA, USA) with fluorescence detection [21]. The ratio of arginine to ADMA was computed to assess NO availability [24].

### 2.4. SCFAs and Receptors

Plasma levels of acetate, propionate, and butyrate were analyzed by GC-MS (QP2010; Shimadzu, Kyoto, Japan) with a flame ionization detector, as detailed in previous studies [22]. Chromatographic separation was achieved with a DB-FFAP column (Agilent Technologies). The sample injection volume was 1 µL, using a split ratio of 5:1, and the injection temperature was consistently maintained at 240 °C. We utilized 2-ethylbutyric acid as the internal standard.

Having established that SCFA receptors regulate BP through their interaction with SCFAs [25], we proceeded to examine the mRNA expression of SCFA receptors by qPCR using SYBR Green. From the kidney cortex of each rat, RNA extraction was conducted. Four SCFA receptors—olfactory receptor 78 (Oflr78), G protein-coupled receptor 41 (GPR41), GPR109A, and GPR43—were the specific focus of our analysis. The obtained results were then normalized to the 18S rRNA (R18S) reference gene. Duplicate runs were carried out for each sample, and a dissociation curve analysis followed all qPCR reactions using primers established earlier [22]. We utilized the comparative threshold cycle method to calculate gene expression.

### 2.5. TMA, TMAO, and DMA

The microbial TMAO metabolic pathway involves the oxygenation of TMA to TMAO, which is further catabolized to dimethylamine (DMA) [26]. As described previously [22], their plasma concentrations were determined using an LC–MS method. LC–MS analyses were performed by electrospray ionization in positive mode using a triple quadrupole mass spectrometry analysis. TMAO, TMA, and DMA were detected in multiple-reaction-monitoring mode, with characteristic precursor–product ion transitions at *m*/*z m*/*z* 76.1→58.1, 60.1→44.1, and *m*/*z* 46.1→30, respectively. Diethylamine served as an internal standard in plasma samples. The mobile phase consisted of a mixture of methanol with 15 mmol/L ammonium formate (20:80, *v*/*v*, phase A) and acetonitrile (phase B), operating at a flow rate of 0.3–1 mL/min.

### 2.6. Microbiome Analysis

DNA from microbial communities was extracted from stool samples, and 16S rRNA sequencing was conducted, following previously published protocols [22]. To prepare a multiplexed SMRTbell library (PacBio, Menlo Park, CA, USA) for sequencing, the bacterial full-length 16S rRNA gene was amplified using barcoded primers. To construct a phylogenetic tree, the QIIME2 pipeline employed the FastTree method, which utilized a collection of sequences representing amplicon sequence variants (ASVs) [27,28].

We calculated two alpha diversity metrics, the Shannon index and Faith’s phylogenetic diversity (PD) index. To assess beta diversity, we employed principal coordinate analysis (PCoA) using unweighted UniFrac distances and Analysis of Similarities (ANOSIM). A linear discriminant analysis effect size (LEfSe) was utilized to detect taxa exhibiting differential abundance.

### 2.7. Statistics

The Shapiro–Wilk normality test was used to determine which data were normally distributed. The data, expressed as means ± standard error of the mean, were analyzed statistically with significance set at *p* < 0.05. One-way ANOVA was utilized for the initial analysis, which was followed by Tukey’s post hoc test for multiple comparisons. In metabolomics analysis for alpha diversity, the Wilcoxon test was utilized to analyze whether the differences in species diversity between two groups were significant. The ANOSIM used an R-value and *p*-value to compare the similarity between groups. Statistical methods were applied to identify species with significant differences in microbial communities between groups. Multiple hypothesis testing and false discovery rate (FDR) analysis were used to assess these differences with an FDR (q-value) < 0.05 considered significant. Furthermore, LEfSe analysis was conducted using the Wilcoxon test.

## 3. Results

### 3.1. Body Weight, BP, and Creatinine Level

**Figure 1:**
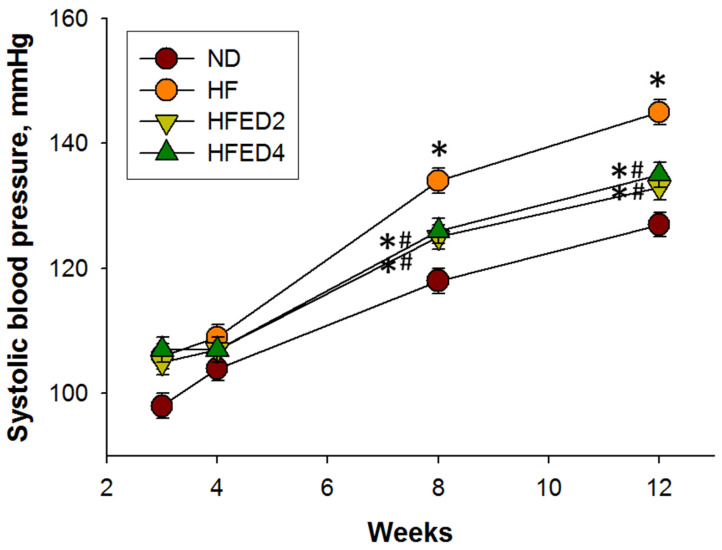
Effects of ED2 and ED4 on systolic blood pressures in offspring from week 3 to 12. Statistical analysis by a one-way ANOVA with Tukey’s post hoc test. * *p* < 0.05 vs. ND; # *p* < 0.05 vs. HF.

The mortality rate was zero in all groups. The ND group exhibited a higher body weight (BW) and kidney weight (KW) compared with the other three groups (all *p* < 0.05), though the ratio of KW to BW remained consistent across all four groups (Table 1). As documented in previous studies [10,12], rat offspring displayed heightened systolic and diastolic BPs due to the maternal consumption of a high-fructose diet by week 12. Figure 1 shows a significant increase in systolic BP in rat offspring starting from week 8 (*p* < 0.05) and continuing through week 12 (*p* < 0.05). However, this increase was notably reduced with the administration of ED2 or ED4 treatment (Both *p* < 0.05). The renal function, indicated by plasma creatinine levels, showed no discernible differences among the four groups (Table 1).

### 3.2. NO Pathway

Given the observed efficacy of ED2 and ED4 in reducing offspring hypertension, our subsequent focus shifted to evaluating the underlying protective mechanisms. Hypertension is associated with low NO levels and elevated oxidative stress [29]. Given the antioxidant effects of resveratrol on NO-mediated vasodilation associated with hypertension improvement [30], we proceeded to investigate whether ED2 or ED4 played a protective role in the NO pathway.

**Figure 2:**
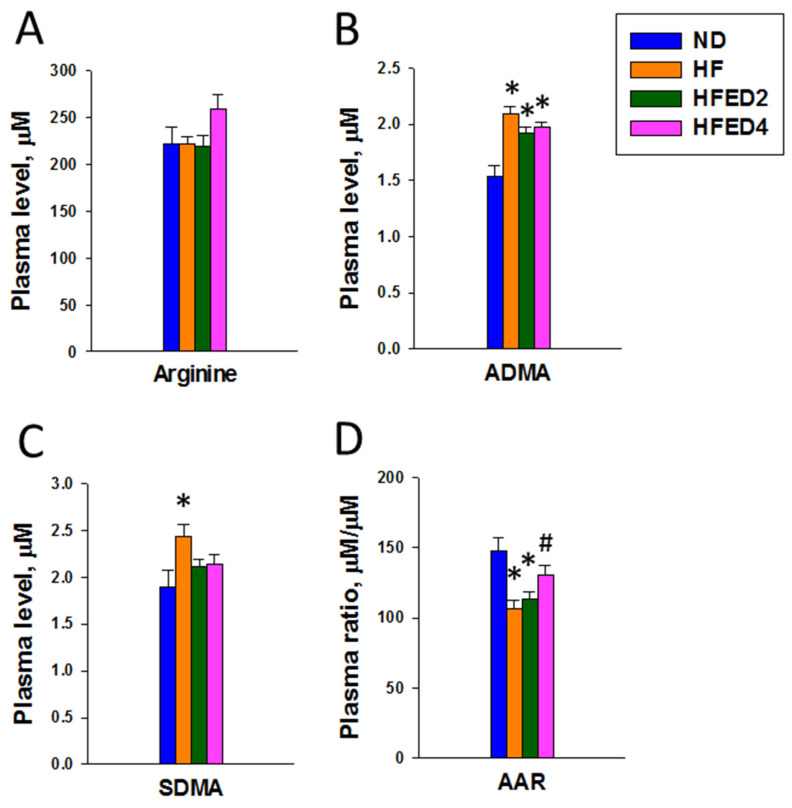
Concentrations of (**A**) arginine, (**B**) asymmetric dimethylarginine (ADMA), (**C**) symmetric dimethylarginine (SDMA), and (**D**) the ratio of arginine to ADMA (AAR) in the plasma. Statistical analysis by a one-way ANOVA with Tukey’s post hoc test. * *p* < 0.05 vs. ND; # *p* < 0.05 vs. HF.

Figure 2A shows that plasma arginine levels are comparable among the four groups. The ND group revealed a lower ADMA concentration compared to the other groups (all *p* < 0.05) (Figure 2B). Furthermore, supplementation with ED2 and ED4 led to a decline in plasma SDMA concentrations in the HFED2 and HFED4 groups compared with the HF group (Figure 2C). Significantly, maternal feeding of a high-fructose diet resulted in a reduction in the AAR, which was an effect prevented by ED4 treatment (Figure 2D).

### 3.3. Gut Microbiota Compositions

**Figure 3:**
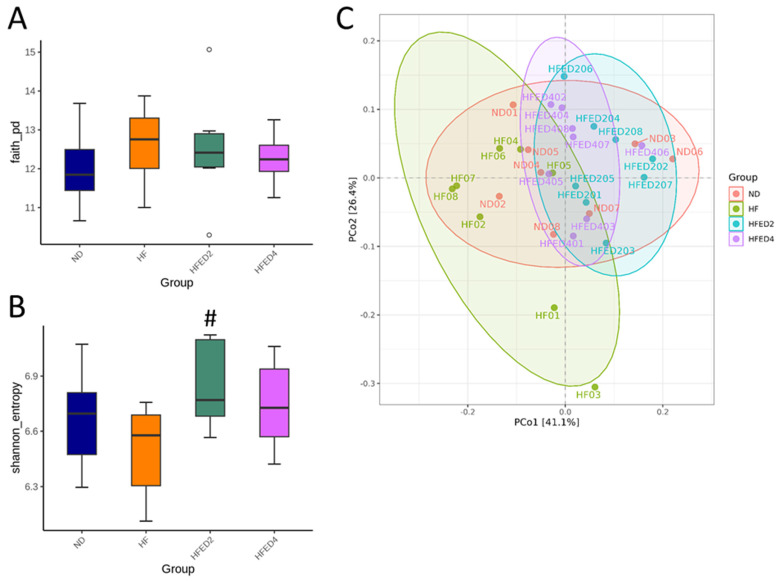
The assessment of gut microbial community α-diversity is demonstrated in (**A**) Faith’s phylogenetic diversity (PD) index and (**B**) the Shannon index. (**C**) Principal coordinate analysis (PCoA) plots were used to visualize beta diversity, with each data point representing one sample and each color corresponding to a different group. # *p* < 0.05 vs. HF.

Conducted as the subsequent experiment, we sought to explore whether the protective effects of ED2 and ED4 were linked to their prebiotic properties and their ability to modify gut microbiota. Illustrated in Figure 3, α-diversity, as indicated by Faith’s PD index (Figure 3A), did not exhibit any significant differences among the four groups. However, the HFED2 group displayed a higher Shannon index compared to the HF group (Figure 3B). In terms of β-diversity, the PCoA analysis demonstrated a clear differentiation of microbial communities among the four groups, forming distinct clusters (Figure 3C). Furthermore, the overall distinction between these grouped communities was assessed through ANOSIM. Statistically significant differences among each group, as revealed by the ANOSIM test (*p* < 0.01), were observed except for the HF group, which did not significantly differ from the ND group (*p* = 0.012).

**Figure 4:**
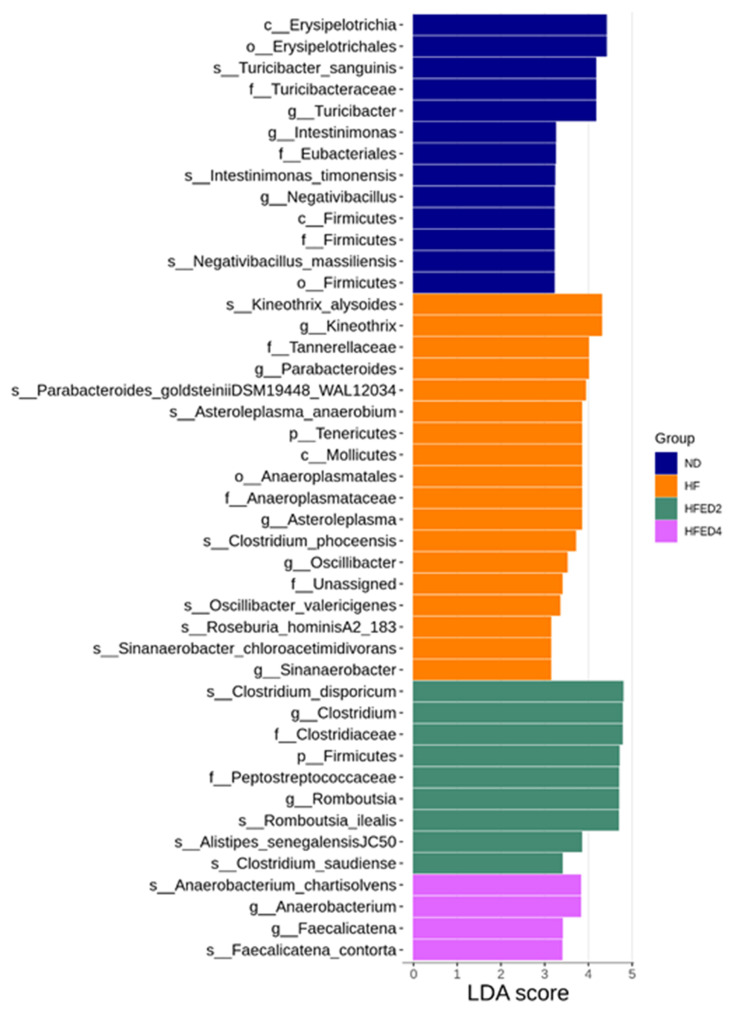
Linear discriminant analysis effect size (LEfSe) was employed to identify taxa that were significantly differentially abundant between groups. Taxa with a linear discriminant analysis (LDA) score greater than 3 were primarily highlighted. Statistical analysis by the Wilcoxon test.

In Figure 4, LEfSe analysis was employed to pinpoint taxonomic differences significantly enriched across the four groups. Particularly, an increase in the proportion of *Clostridium* and *Romboutsia* genera was detected after ED2 treatment. Conversely, treatment with ED4 led to an elevated proportion of *Anaerobacterium* and *Faecalicatena*.

**Figure 5:**
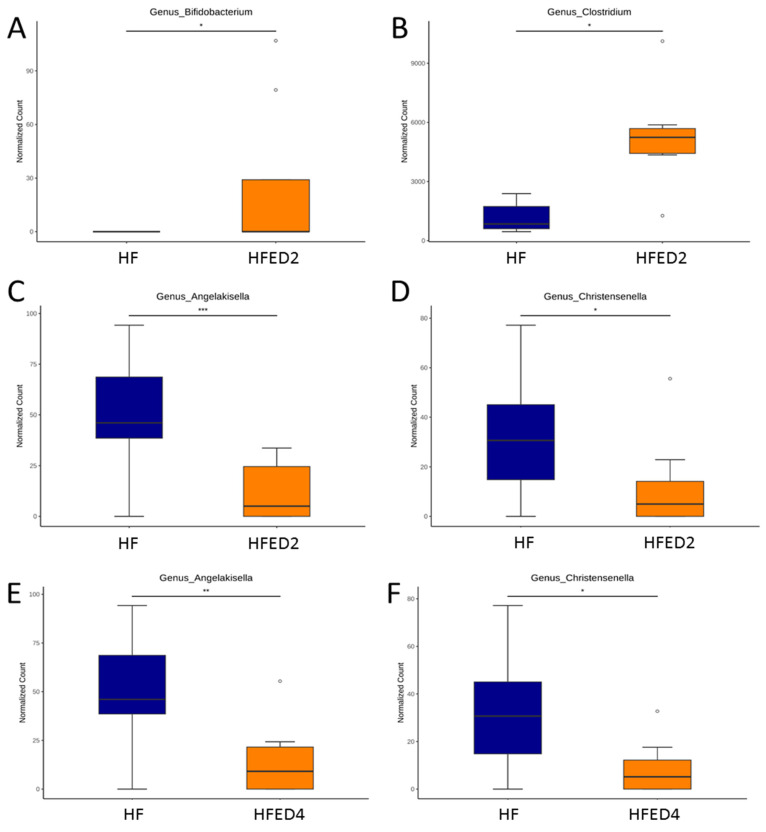
Genus-based comparison between the HF and HFED2 group showing relative abundance of (**A**) *Bifidobacterium*, (**B**) *Clostridium*, (**C**) *Angelakisella*, and (**D**) *Christensenella*. Genus-based comparison between the HF and HFED4 group showing relative abundance of (**E**) *Angelakisella* and (**F**) *Christensenella*. The dots represent the outliers. Statistical analysis by using *p*-values adjusted for multiple comparisons with the false discovery rate (FDR) method. * *p* < 0.05; ** *p* < 0.01; *** *p* < 0.005.

Subsequently, our investigation delved into a genus-based comparison between the HF and HFED2 groups and between the HF and HFED4 groups. Notably, ED2 led to an increase in the genera *Bifidobacterium* (*p* < 0.05) and *Clostridium* (*p* < 0.05) compared to the HF group (Figure 5A,B). Furthermore, both ED2 and ED4 demonstrated a reduction in the abundance of *Angelakisella* (*p* < 0.005) and *Christensenella* (*p* < 0.05) (Figure 5C–F).

### 3.4. SCFAs and Their Receptors

**Figure 6:**
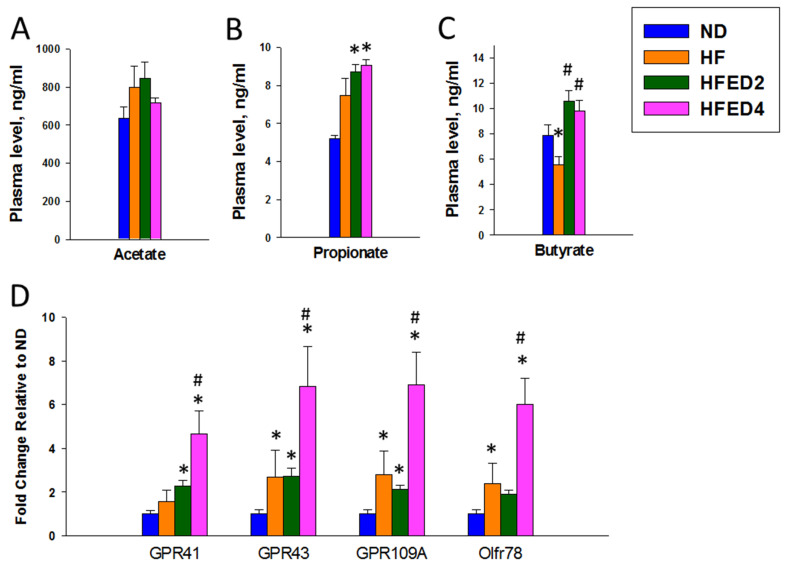
Plasma concentrations of (**A**) acetate, (**B**) propionate, (**C**) butyrate, and (**D**) mRNA expression of their receptors in rat kidneys, including G protein-coupled receptor 41 (GPR41), GPR43, GPR109A, and olfactory receptor 78 (Oflr78). Statistical analysis by a one-way ANOVA with Tukey’s post hoc test. * *p* < 0.05 vs. ND; # *p* < 0.05 vs. HF.

Examining the most abundant gut microbial metabolites, SCFAs, we proceeded to determine their concentrations in the plasma among the four groups. As depicted in Figure 6A, plasma levels of acetate did not exhibit significant differences among the four groups. However, both ED2 and ED4 led to increases in plasma concentrations of propionate compared to the ND group (Figure 6B). Notably, a maternal diet high in fructose resulted in a reduction in plasma butyrate levels (*p* < 0.05), which is an effect counteracted by the administration of ED2 or ED4 treatment (Figure 6C).

In further analyzing SCFA receptor expression in the offspring’s kidneys, we considered their role in regulating blood pressure through interaction with SCFAs [21]. Figure 6D illustrates that a maternal diet high in fructose induced an increase in the expression of GPR43, GPR109A, and Olfr78 compared with those in the ND group. Additionally, the mRNA expression of GPR41, GPR43, and GPR109A was greater in the HFED2 group than in the ND group. Furthermore, the HFED4 group exhibited the highest expression of all four SCFA receptors among the groups (Figure 6D) (all *p* < 0.05).

### 3.5. TMAO Metabolic Pathway

Turning our attention to the TMAO metabolic pathway, which has been linked to the positive effects of resveratrol [21], we investigated its status. As outlined in Table 2, plasma levels of TMA did not exhibit significant differences among the four groups. ED2 was linked to a reduction in plasma concentrations of DMA (*p* < 0.05), while ED4 led to a decrease in TMAO (*p* < 0.05).

## 4. Discussion

Exploring the potential of purified RBE monomers, ED2 and ED4, to alleviate hypertension in a maternal high-fructose diet model is a pioneering aspect of this study. Our major findings are summarized as follows: (1) offspring hypertension, programmed by a maternal diet high in fructose, can be alleviated through perinatal supplementation with ED2 or ED4; (2) the beneficial effects of ED4 are associated with an increase in NO availability, which is evident in an elevated AAR; (3) ED2 and ED4 induce distinct alterations in gut microbial compositions with only ED2 showing an increase in α-diversity; (4) the protective influence of ED2 is linked to an increased abundance of the genera *Bifidobacterium* and *Clostridium* coupled with a decrease in *Angelakisella* and *Christensenella*; (5) ED4 shields rat offspring from hypertension, which is accompanied by elevated concentrations of propionate, butyrate, and their SCFA receptors, and a reduction in TMAO.

This study supports prior research highlighting the health benefits of perinatal resveratrol derivatives for offspring [31,32], contributing novel insights into the development of ED2 and ED4 for their impact on offspring hypertension with developmental origins. Significantly, both purified RBE monomers, ED2 and ED4, exhibit similar protective effects against hypertension programmed by maternal diet high in fructose, paralleling the reported benefits of resveratrol [13]. Notably, it is essential to highlight that ED2 and ED4 both serve a dual role as natural antioxidants and prebiotics similar to resveratrol [33,34].

Building upon prior work demonstrating the protective effects of supplementing RBE mixture during gestation and lactation against hypertension in progeny exposed to di-2-ethylhexylphthalate (DEHP) [35], this study indicates that both ED2 and ED4 yield comparable BP-lowering effects despite each displaying differential protective mechanisms. Firstly, the observed benefits of both RBE derivatives are linked to the NO pathway. Given that elevated SDMA levels contributing to NO deficiency play a role in hypertension [36], the reduction in SDMA by ED2 and ED4 was associated with their BP-lowering effects. Moreover, ED4 demonstrates the ability to elevate the AAR, suggesting an enhancement of the NO pathway.

Another protective mechanism against offspring hypertension may stem from the prebiotic properties of ED2 and ED4. Although both RBE derivatives exhibit no difference in α-diversity, ED2 increases β-diversity. ED2 supplementation increases the abundance of beneficial microbes, including *Romboutsia, Bifidobacterium*, and *Clostridium* [37], suggesting a connection between the beneficial effects of ED2 and an increase in these probiotics.

Corroborating with research in hypertensive individuals [38,39,40], our study reveals negative associations between BP and the proportion of the genera *Clostridium* and *Romboutsia*, while positive correlations exist with the proportion of *Angelakisella* and *Christensenella*. ED2 and ED4 reduce the abundance of *Angelakisella* and *Christensenella*, while *Clostridium* and *Romboutsia* are enriched by ED2 treatment, suggesting a potential connection between the beneficial actions of ED2 and ED4 and their abilities to alter BP-associated taxa.

Not only altered microbiota composition but also microbial metabolites are associated with the protective effects of ED2 and ED4 against offspring hypertension. Both derivatives enhance propionate production and restore the reduction in butyrate induced by maternal high-fructose intake. The findings on SCFAs are consistent with earlier studies that highlight the BP-lowering effects of SCFAs [20]. In line with prior work revealing that supplementation with RBEs can lead to an increase in plasma butyrate concentration [41], our data demonstrate this trend.

The role of SCFAs in BP regulation is well established with their binding to different receptors exerting opposing effects [20]. Specifically, the activation of GPR41 has been associated with BP reduction, which is a response that can be offset by GPR43 and Olfr78, leading to hypertension [42]. In our study, we observed that ED2 not only increased butyrate levels but also concurrently upregulated the expression of GPR 41 and GPR 43. Additionally, ED4 exhibited a significant increase in all four SCFA receptors. The role of ED2 and ED4 in modulating SCFAs and their receptors and how these effects contribute to their beneficial impact on offspring hypertension requires further investigation.

Another potential positive mechanism underlying the protective effects of ED2 and ED4 against offspring hypertension may lie in their ability to regulate TMAO metabolism. Our previous in vitro study demonstrated that an RBE mixture could reduce TMA formation, and both ED2 and ED4 effectively decreased TMAO levels in TMA-induced HepG2 cell lines [23]. In the present study, ED4 reduced TMAO levels, while ED2 decreased its metabolite DMA, suggesting their involvement in the TMAO metabolic pathway. The participation of various bacteria in TMA formation has been demonstrated, covering *Anaerococcus*, *Escherichia*, *Edwardsiella*, *Providencia*, *Proteus*, etc. [43]. Nevertheless, their abundance showed negligible changes with ED2 or ED4 treatment. In contrast, certain gut microbes, such as *Bifidobacterium* [44], possess the capability to convert TMAO back into TMA. Notably, we observed an increased proportion of *Bifidobacterium* with ED2 treatment, although it did not affect the levels of either TMAO or TMA. Further research is essential to determine whether bacteria involved in both TMA synthesis and degradation contribute to the protective effects of the RBE-mediated TMAO metabolic pathway.

Given that excessive fructose intake is a modifiable risk factor, educating pregnant and lactating women to limit their consumption of high-fructose diets is crucial for preventing hypertension in their offspring. Despite this, current fructose intake among pregnant women still exceeds recommended levels [5]. Additionally, other early-life factors, such as maternal illness, exposure to environmental chemicals, and medication use, also contribute to the developmental programming of hypertension [45]. Therefore, further research is needed to explore the protective effects of ED2/ED4 in various animal models of hypertension with developmental origins to support future clinical applications.

While this study provides valuable insights, it has some limitations. Firstly, the doses of ED2 and ED4 tested may not fully reveal their dose-dependent effects, suggesting the need for future studies using a range of doses. Given that some human studies have reported negative results regarding resveratrol [46], it is urgent to evaluate the efficacy, bioavailability, and pharmacodynamics of these two RBE components to determine appropriate dosages for human use. Next, further research is needed to identify specific bacterial strains that interact with SCFAs and TMAO and to evaluate microbial function in order to clarify the contributory role of the gut microbiota. Thirdly, the causal relationship between gut microbiota structures, microbial metabolites, and the beneficial actions of ED2/ED4 necessitates further exploration. Although ANOSIM analysis indicated statistically significant differences among most groups, the PCoA analysis revealed some overlap among them. This overlap may reflect the underlying ecological similarities between the groups. Additionally, other factors influencing the clustering, such as unaccounted variables or within-group variability, might also contribute to this observed overlap. Lastly, the potential sex-dependency or model-specific nature of the antihypertensive actions of ED2/ED4 should be further investigated through additional studies involving both sexes and alternative developmental programming models of hypertension.

## 5. Conclusions

In conclusion, supplementation with the purified RBE monomers ED2 and ED4 shows beneficial effects in a model of offspring hypertension induced by a maternal diet high in fructose. These effects are associated with improved NO availability, increased levels of SCFAs, alterations in the TMAO metabolic pathway, and modifications in the gut microbiota. The newly synthesized resveratrol-derived compounds hold promise for improving offspring hypertension and broadening the clinical applications of resveratrol-based natural products.

## Figures and Tables

**Table 1:** Weights, blood pressure, and creatinine level.

Groups	ND	HF	HFED2	HFED4
Body weight (BW) (g)	298 ± 37	273 ± 10 *	264 ± 7 *	263 ± 12 *
Left kidney weight (KW) (g)	1.31 ± 0.14	1.2 ± 0.05 *	1.17 ± 0.02 *	1.22 ± 0.05 *
The ratio of KW to BW (g/kg)	4.4 ± 0.2	4.4 ± 0.1	4.5 ± 0.1	4.7 ± 0.1
Systolic blood pressure (mmHg)	127 ± 1	145 ± 1 *	133 ± 1 *#	135 ± 1 *#
Diastolic blood pressure (mmHg)	88 ± 1	96 ± 2 *	87 ± 3 #	96 ± 2 *
Creatinine (μM)	14.5 ± 0.9	14.6 ± 1.4	12.9 ± 0.4	12.6 ± 0.6

Statistical analysis by a one-way ANOVA with Tukey’s post hoc test. * *p* < 0.05 vs. ND; # *p* < 0.05 vs. HF.

**Table 2:** Plasma concentrations of trimethylamine (TMA), trimethylamine-*N*-oxide (TMAO), and dimethylamine (DMA).

Groups	ND	HF	HFED2	HFED4
TMA, ng/mL	3.06 ± 0.48	5.94 ± 1.86	5.06 ± 1.06	3.99 ± 1.13
TMAO, ng/mL	283.6 ± 19.7	266.9 ± 8.11	283.9 ± 19.6	219.8 ± 13.6 *#
DMA, ng/mL	113.5 ± 9.1	135.8 ± 5.8	106.2 ± 8.4 #	130.3 ± 11.1

Statistical analysis by a one-way ANOVA with Tukey’s post hoc test. * *p* < 0.05 vs. ND; # *p* < 0.05 vs. HF.

## Data Availability

Data are contained within the article.
